# Supplementary material for: Cloud BioLinux: pre-configured and on-demand bioinformatics computing for the genomics community
Source: BMC Bioinformatics. 2012 Mar 19;13:42. doi: 10.1186/1471-2105-13-42 (PMC3372431; doi:10.1186/1471-2105-13-42)
Supplement: Additional file 1 — Supplementary 1 Cloud BioLinux software documentation in the form of a mini, self-contained website. Users need to download and uncompress the .zip file, and open through a web browser the "index.html" file available on the main directory. (ZIP 1823 kb). [file 1471-2105-13-42-S1.ZIP › Cloud-BioLinux-Package-Documentation/docs/denoiser.html]

Bio-Linux Software Documentation Pages

Back to search form

## denoiser

|  |  |
| --- | --- |
| Name | denoiser |
| Description | **Denoiser** is designed to address issues of noise in pyrosequencing data. Denoiser is a heuristic variant of PyroNoise. Developers of denoiser report a good agreement with PyroNoise on several test datasets.  **Denoiser** uses three clustering steps:  1. cluster perfect reads on the sequence level and get an initial    abundance distribution of the common OTU 2. cluster near-perfect reads to the common OTUs using flowgram    alignments and a strict clustering threshold 3. cluster bad reads not previously clustered using a relaxed    threshold   To start up the denoiser environment on Bio-Linux, you need to type: `denoiser` If you wish to use the sffinfo command referred to in the documentation, you will need to install gsAssembler from Roche on your machine.  A different version of the denoiser.py script is run from within the qiime environment than from within the denoiser environment. To run denoiser from within the qiime environment, exit from the denoiser environment if you are in it, and then type `qiime` on the command line. |
| Homepage | http://www.microbio.me/denoiser/ |
| Remote Documentation |  |
